# Evaluations of the sum-score-based and item response theory-based tests of group mean differences under various simulation conditions

**DOI:** 10.1177/09622802211043263

**Published:** 2021-10-07

**Authors:** Mian Wang, Bryce B. Reeve

**Affiliations:** 1 Lineberger Comprehensive Cancer Center, 2331 University of North Carolina at Chapel Hill, Carrboro, NC, USA; 2 Department of Population Health Sciences, 3065 Duke University School of Medicine, Durham, NC, USA

**Keywords:** Group mean comparison, sum-score, expected-a-posteriori score, item response theory, decision tree

## Abstract

The use of patient-reported outcomes measures is gaining popularity in clinical trials for comparing patient groups. Such comparisons typically focus on the differences in group means and are carried out using either a traditional sum-score-based approach or item response theory (IRT)-based approaches. Several simulation studies have evaluated different group mean comparison approaches in the past, but the performance of these approaches remained unknown under certain uninvestigated conditions (e.g. under the impact of differential item functioning (DIF)). By incorporating some of the uninvestigated simulation features, the current study examines Type I error, statistical power, and effect size estimation accuracy associated with group mean comparisons using simple sum scores, IRT model likelihood ratio tests, and IRT expected-a-posteriori scores. Manipulated features include sample size per group, number of items, number of response categories, strength of discrimination parameters, location of thresholds, impact of DIF, and presence of missing data. Results are summarized and visualized using decision trees.

## Introduction

In the past two decades, the importance of patient-reported outcomes (PROs) has been well recognized in the medical and healthcare research community, and an increasing number of clinical trials have included endpoints utilizing PRO instruments.^1,2^ Typically, a PRO instrument consists of multiple survey questions that are intended to assess patients’ levels on a latent construct that is often not directly observable (e.g. post-treatment life satisfaction). Based on such an assessment, sample means can be estimated for predefined patient groups in a clinical trial, and thus group comparisons can be carried out.

The traditional group mean comparison approach calculates individual scores and subsequently test mean differences between groups while adjusting for variation. For example, a simple method is to sum up all numeric item responses for each person within the respective groups and then run a general linear model (typically, a *t*-test) to compare the group means estimated from the sum scores.

More recently, item response theory (IRT) models have gained popularity in the PRO field,^3,4^ and IRT-model-based group mean comparisons are not uncommon in applications.^5,6^ Under an IRT model, the probability of observing each item response is expressed as a function of both item parameters and person scores on a latent factor (i.e. the construct of interest). When multiple patient groups are involved, a reference group is selected to set the common metric with its mean fixed at zero and variance fixed at one, while all other groups’ factor means and variances can be estimated alongside the unknown item parameters during model calibration. Therefore, unlike the traditional mean comparison approach, without calculating individual scores, the IRT-model approach can directly compare groups using either a Wald test (i.e. the difference in factor means divided by the standard error) or a likelihood ratio test (i.e. a chi-square difference test comparing two models with and without an equality constraint placed on factor means).

Furthermore, when item parameters have been pre-calibrated in large representative samples using IRT techniques, direct calculations of the expected-a-posteriori (EAP) scores can substitute for the IRT model calibration step, followed by group mean and variance estimation and group comparison procedures that are similar to the traditional approach. EAP scoring is also widely adopted in PRO research.^7–9^

To the best of our knowledge, only a limited number of past simulation studies have directly compared the traditional sum-score approach against IRT-based (or more generally, latent-variable-based) approaches in terms of their statistical power and Type I error associated with group mean comparisons.^10–13^ Overall, researchers found that these approaches showed comparable power and Type I error in most of the studied conditions unless missing data were involved (which resulted in reduced power for the sum-score approach with listwise deletion) or a short measure was used (which resulted in reduced power for the IRT-model approach). However, a few things were missing in previous studies.

First, none of the abovementioned studies has directly tested the impact of differential item functioning (DIF) or the lack of measurement invariance on group mean comparisons. DIF occurs when the probability of choosing a specific item response differs between groups even if those groups are matched on the underlying construct.^14^ Under the impact of uncontrolled DIF, scores calculated for the PRO measure are biased and we are unable to establish a fair comparison between the affected groups. Given that DIF is a prevalent phenomenon in PRO research,^15–18^ it is imperative to understand its impact on group mean comparisons. Due to DIF's potential significant biasing effects on estimating sum scores and latent trait scores as found in past simulation research,^19^ we hypothesize that group mean comparison results will be distorted by the introduction of DIF items and whether DIF prescreening was performed as a remedy, and we also expect such effects to interact with other manipulated features including the type of group mean comparison method being implemented.

Second, none of the abovementioned studies has examined conditions using items that follow the two-parameter logistic IRT model (which is equivalent to a two-category graded response model),^20^ and no direct comparison between the dichotomous and polytomous item conditions has been conducted. Past research has suggested that person scores estimated using dichotomous items have a much lower correlation with the true data-generating scores when compared to scores estimated using polytomous items with four or more response categories.^21^ Thus, we hypothesize that group mean comparisons will yield more accurate results when items are polytomous with five response categories, and such an effect will likely interact with other manipulated features.

Lastly, none of the abovementioned studies has manipulated the magnitude of an items’ discrimination (high vs. low) and the locations of items thresholds (diverse vs. homogeneous within the item set). Past research has shown that having higher discrimination parameters also resulted in better estimation of person scores and better psychometric properties overall.^21^ Thus, we hypothesize that group mean comparisons will yield more accurate results when items are highly discriminating. We also hypothesize that having items with a diverse set of thresholds (i.e. more spread out on the latent factor) is more beneficial for person score estimation than having items with homogeneous thresholds because items under the former condition can better differentiate a wider range of people at different locations on the latent factor. In addition, we expect that other manipulated features would interact with the effects of discrimination and thresholds parameters on group mean comparisons.

Therefore, the current study aims to revisit and reevaluate the performance of the different group mean comparison approaches by incorporating the above features that have not been investigated in the past.

## Method

For the current simulation study, we manipulated the following features: sample size per group (100 or 400), number of items (5 or 20), strength of item discrimination parameters (high or low), number of response categories (dichotomous or five-category polytomous), location of item thresholds (homogeneous or diverse), impact of DIF or DIF-free, and presence of missing data (complete data or 30% cases with missing at random data). For the current study, we would like to obtain a broader picture of how different features interact and offer some guidance on future research. Hence, we manipulated more features than what we had in the main research questions, including some features that have been evaluated in past research (i.e. sample size, number of items, and missing data).

In addition, the true group mean difference was set either at zero for Type I error evaluation or at 0.5 (in *z*-score metric) for power evaluation. During the simulation, 450 replications were carried out for each simulation condition. The simulation process was run by a high-performance computing server using 80 parallel threads.

### The data generation phase

True scores for the simulated respondents were generated first, followed by random draws of item parameters and DIF effects (if any). Using the simulated true scores and item parameters, item response data were then generated under the IRT graded response model using the ‘mirt’ package in R.^20,22^ When needed, missing responses were simulated using the ‘mice’ package in R.^23^ More details regarding each step of the data generation phase are described next.

#### The IRT graded response model

Both polytomous and dichotomous data were generated using Samejima's graded response model, which is explained below.^20^ Let $u_{ij}\in \{0,1,\ldots ,V-1\}$ be the item response of person *i* on item *j* with *V* number of strictly ordered categories, under the unidimensional (i.e. one latent factor) graded response model, we have the following category response functions for the cumulative probabilities of observing a response falling in a specific category or higher, given the person's true score on the latent construct θ and item parameters $a_{j}$ and $b_{\;j,*}$ .

(1) $$P(u_{ij}\geq 1)=\frac{\exp\;\{Da_{j}(\theta_{i}-b_{\;j,1})\}}{1+\exp\;\{Da_{j}(\theta_{i}-b_{\;j,1})\}}$$

⋮

(2) $$P(u_{ij}\geq V-1)=\frac{\exp\;\{Da_{j}(\theta_{i}-b_{\;j,V-1})\}}{1+\exp\;\{Da_{j}(\theta_{i}-b_{\;j,V-1})\}}$$

In the above, $b_{\;j,1},\ldots ,b_{\;j,V-1}$ are the thresholds that determine the steepest locations of the probability curves where the probability equals 0.5, and $a_{j}$ is the discrimination parameter which represents the slope of the curve at locations of the thresholds. *D* = 1.7 is a scaling constant which only applies when converting a discrimination parameter estimated under the logistic model to its counterpart in the normal ogive scale. Consequently, the item response function for responding in each category is calculated as the difference between two consecutive category response functions. For example, the item response function for observing a response in category 2 is

(3) $$P(u_{ij}=2)=P(u_{ij}\geq 2)-P(u_{ij}\geq 3)$$

As special cases on the boundaries, the first and the Vth categories are respectively defined as $P(u_{ij}=0)=1-P(u_{ij}\geq 1)$ and $P(u_{ij}=V-1)=P(u_{ij}=V-1)$ , because $P(u_{ij}\geq 0)=1$ and $P(u_{ij}\geq V)=0$ by definitions of probability.

Please note that the above expressions can only be used for data generation when the true scores and item parameters are known a priori. During the calibration of an IRT model, however, the item parameters are typically unknown and need to be estimated. Therefore, the following marginal likelihood function is maximized to obtain the unknown parameters ( β ), based on data collected from *N* subjects and *J* items.

(4) $$L(\beta |\mathrm{data})=\overset{N}{\underset{i=1}{\prod }}\;\left[\overset{+\infty }{\underset{-\infty }{\int }}\;\left(\overset{J}{\underset{\;j=1}{\prod }}\;P(u_{ij}|\beta )\right)f(\theta )d(\theta )\right]$$

In the above, $\prod_{\;j=1}^{J}P(u_{ij}|\beta )$ is the product of all item response functions associated with person *i*'s response pattern conditioning on the unknown parameters, and f(θ) is the distribution of the latent factor. Under the conventional IRT assumptions, f(θ) is normally distributed, all items are locally independent (i.e. no/little covariation other than through the latent factor), and all respondents are independent.

#### True scores

Individual scores for the reference group were randomly drawn from a standard normal distribution ( $\theta_{R}∼N(0,\;1)$ ), whereas scores for the focal group were randomly drawn from a normal distribution with its mean fixed at one of the two manipulated values ( ${\bar{\theta }}_{F}\in \{0,\;0.5\}$ ) and SD fixed at 1. Hence, the focal group mean also represented the effect size for the true difference between groups.

#### Item parameters

The distributions from which the item parameters were randomly drawn were informed by summaries of item parameters reported in past empirical and simulation research studies.^3,24–26^ Specifically for the empirically calibrated items, the six studies cited in Wang and Woods^24^ reported parameters for 598 dichotomous items, and the first wave of item calibrations under the Patient-Reported Outcomes Measurement Information System® (PROMIS®) initiative^3^ reported more than 600 five-category polytomous items covering both physical and psychological domains. Necessary alterations were also made in the current study to ensure that the true item parameters did not end up with extreme or unrealistic values (e.g. negative item discriminations).

Discrimination parameters were randomly drawn from a uniform distribution between 1.7 and 3.5 for the high-discrimination conditions (given our logistic IRT models, these values would translate to standardized factor loadings between 0.71 and 0.90 under factor analysis models),^27^ and between 0.5 and 1.0 for the low-discrimination conditions (i.e. factor loadings between 0.28 and 0.51).

For dichotomous items, thresholds were randomly drawn from a normal distribution ( $b_{j}∼N(0.1,\;1.3^{2})$ ) with truncations at −2.5 and 2.4. For every polytomous item with five response categories, the first threshold was randomly drawn from a normal distribution ( $b_{\;j1}∼N(-1,\;1)$ ) with truncations at −3.5 and 0.5, and the remaining three thresholds were derived by successively adding values randomly drawn from a normal distribution ( $b_{\Delta }∼N(0.8,\;0.2^{2})$ ) with truncations at 0.4 and 1.2. Effectively, the largest thresholds for the polytomous items were bounded between 0.1 and 4.1.

Respectively for the dichotomous and polytomous types, parameters of 10,000 items were generated and saved in a table, from which the data-generating items were randomly chosen during each simulation replication. Under the homogeneous thresholds condition, a benchmark item was drawn first, and then the remaining items were drawn so that their thresholds were within a ±0.25 range of the benchmark items. If there were an insufficient number of items meeting the above requirement, the simulation function would skip the replication and restart another. In contrast, under the diverse thresholds condition, all items were simultaneously drawn without any restrictions on their thresholds.

#### DIF effects

Under the DIF-free condition, the item parameters used to generate item responses were identical for both groups. Under the DIF condition, focal group item parameters were modified so that 40% of the items had lower discriminations but higher thresholds than their counterparts in the reference group, which mimicked a situation under which the focal group is disadvantaged. Differences in the discrimination parameters were randomly drawn from a uniform distribution between 0 and 0.3 (the 0.3 upper bound prevented negative values under the low-discrimination conditions), while differences in the thresholds were drawn from a uniform distribution between 0.5 and 0.7. Post-simulation summary revealed that the expected test score standardized difference ranged between −0.77 and 0.25 (mean = −0.22 and interquartile range between −0.25 and −0.20), suggesting that the majority of replications under the DIF condition suffered a small size DIF impact at the test level (i.e. differential test functioning (DTF); see Equation 10 in Meade's article^28^) when compared to Cohen's *d* standards.

It is worth pointing out that, even though we purposely simulated the DIF effects to put the focal group at a disadvantage (i.e. expecting a negative DTF effect size), we found the opposite effect within 148 replications (approximately 0.1% of the total). Further investigations found that over 85% of these replications came from conditions where the true focal group mean was set at zero and the discrimination parameters were low. These observations suggested that the unexpected positive DTF phenomenon was likely due to the combination of having non-uniform DIF items (each group is disadvantaged in different areas on the latent factor scale) and a large concentration of focal group individuals in the more advantaged areas because the focal group sample was used for our DTF effect size calculation. Nonetheless, the final results and conclusions remained unchanged with or without these special cases.

#### Missing data

For replications with missing data, item responses on 20% of randomly chosen items were removed for 30% of the subjects. The removal procedure followed the missing at random mechanism under which the probability of a missing response could be fully explained by the observed/non-missing responses on the other items (i.e. the remaining 80% of items that were not chosen to have missing data).

### The statistical testing phase

For each replication, three different mean comparison approaches (sum-score, IRT-model, and IRT-EAP) were carried out, with or without DIF prescreening, to compare the means of the reference and focal groups. Effect sizes for group mean differences were also quantified and compared to Cohen's *d* standards under each approach. Details regarding the testing procedures are described next.

#### The sum-score approach

Respectively for the reference and focal groups, we obtained the sum scores by adding up all numeric responses for each simulated respondent, while omitting cases with missing values (i.e. listwise deletion). A two-sided independent-samples Welch's *t*-test was then carried out to compare the reference and the focal groups without assuming equal population variances.^29^ A test statistic associated with a probability less than the nominal alpha level of 0.05 would be flagged as statistically significant. In addition, an effect size was calculated by dividing the difference in group means by a pooled standard deviation.

#### The IRT-model approach

For this approach, two unidimensional graded response IRT models were fit to the data. In the first model, the two groups were linked on the same metric by constraining all item parameters equal between groups (i.e. assumed full measurement invariance), and the focal group factor mean and variance were freely estimated. Next, a more restricted model was fit to the data by adding an equality constraint on the two-factor means on top of the previous model. The unknown parameters (e.g. item parameters, and focal group mean and variance) in the models were obtained by maximizing the marginal likelihood of the observed data.^30^ To compare means of the two groups, the significance of the equality constraint between the two-factor means was tested using a likelihood ratio test based on the ratio between the likelihoods of the general model ( $L_{G}$ ) and the more restrictive/simpler model ( $L_{S}$ ), the test statistic is given by

(5) $$\chi_{LRT}^{2}=-2\ln\;\left(\frac{L_{S}}{L_{G}}\right)=-2(\ln\;L_{S}-\ln\;L_{G})=\chi_{S}^{2}-\chi_{G}^{2}$$

The test statistic is asymptotically $\chi^{2}$ distributed with degrees of freedom equal to the difference in free parameters between the two nested models. In the current study, the degrees of freedom always equaled 1, because we were testing only one equality constraint between the two factor means. Also, because the reference group factor was fixed as standard normal (mean = 0 and SD = 1) under the IRT model assumption, we were essentially testing whether the focal group mean was significantly different from zero. Significant results (*p* < 0.05) were flagged, and effect sizes were calculated in a similar way as before using the estimated focal group factor mean and variance.

#### The IRT-EAP approach

EAP scores were calculated by taking the expected value of a posterior distribution under IRT. For a given response pattern ( u ), the posterior distribution is constructed by following the Bayes’ theorem.

(6) $$P(\theta |u)=\frac{P(u|\theta )*f(\theta )}{\int_{-\infty }^{+\infty }\;[P(u|\theta )*f(\theta )]d\theta }$$

In the above, $P(u|\theta )=\prod_{\;j=1}^{J}P(u_{j})$ which is the probability of observing response pattern u conditioning on the underlying factor, and f(θ) is the presumed normal distribution of the underlying factor with mean = 0 and SD = 1 (i.e. the prior distribution). Item parameters are assumed to be known parameters during EAP calculations since the IRT-EAP approach typically utilizes a set of item parameters pre-calibrated in large representative samples. A formal introduction on EAP score calculations can be found in Chapter 7 of the book by Embretson and Reise.^14^

Please note that, in the current study, the reference group item parameters were used for constructing P(u|θ) in both the reference and focal groups. When the DIF prescreening procedure was skipped or DIF was not found, the whole set of reference group item parameters were used for EAP score calculations; when DIF has been detected, we would only use the subset of items with nonsignificant DIF for EAP score calculations.

In addition, because a set of known (or well-calibrated) item parameters is required for constructing the item response functions while using the IRT-EAP approach, we followed a similar procedure implemented in past simulation studies by adopting a modified version of the true data-generating item parameters for EAP score calculations.^10^ Specifically, we assumed that the entire item bank had been pre-calibrated with small errors (randomly jiggling the true reference group parameters within a ±0.2 range).

Once the EAP scores were obtained, group means were compared using the same Welch's *t*-test and effect size calculation procedures as the sum-score approach. In comparison to the IRT-model approach, the main advantage of the IRT-EAP approach is its computational efficiency (i.e. does not require fitting IRT models ad hoc). However, the IRT-EAP approach's main disadvantage is that it typically requires pre-calibration of the item parameters in large representative samples for high precision.

#### DIF prescreening

Given that DIF was manipulated and group mean comparisons could be biased under DIF impact, the above three mean comparison approaches were repeated after all items had been prescreened for DIF. For convenience, we employed the IRT likelihood ratio test for both anchor selection and DIF testing during the prescreening process.^31^ By following the steps recommended in published DIF studies,^24,25,32^ we first applied the all-others-as-anchors DIF testing method combined with a rank-based criterion to select up to 20% of items as presumed group-invariant anchoring items. Specifically, in the first step, every item was sequentially tested for DIF while treating all other items as group-invariant anchors whose parameters were fully constrained equal between groups. Next, the top 20% of items of those not showing significant DIF with the largest discrimination parameters were selected as anchors; otherwise, when there is an insufficient number of items showing nonsignificant DIF, the top 20% of items with the smallest test statistics were selected. Once the anchors were selected, we retested the non-anchors for DIF while fixing parameters of the anchors equal between groups. Finally, only the anchors selected in the first step and additional items identified with nonsignificant DIF during the retests were used for calculating the sum and EAP scores, as well as for linking the two groups in the IRT-model approach. Please note that items showing significant DIF effects remained in the IRT-model approach with freely estimated parameters, whereas sum and EAP score calculations completely omitted DIF items.

### The performance evaluation phase

#### Evaluation criteria

Type I error was calculated for conditions that had a true focal group mean of zero which was identical to the reference group mean. Specifically, the proportion of replications that falsely yielded significant results from the *t*-tests or likelihood ratio tests were summarized within the respective simulation conditions. Similarly, statistical power was calculated for each condition that was associated with a non-zero focal group mean. To quantify bias associated with the effect size estimates, mean absolute error (MAE) was computed and summarized respectively for all conditions regardless of the focal group mean. During post-simulation analyses, each summary table was also expanded by crossing with the three mean comparison approaches (sum-score/IRT-model/IRT-EAP) and the two DIF handling procedures (non-prescreened/prescreened) to create additional unique conditions.

#### Evaluation method

To show the effects of the newly incorporated features and their interactions with other features on group mean comparisons, a nonparametric decision tree model was fit to each of the three expanded summary tables, treating each evaluation criterion as the outcome variable and all available features as predictor variables. Utilizing a tree plot based on simple splitting rules, we can quickly identify the most influential features and easily demonstrate their interactions, which is sometimes difficult to discern in a large descriptive table or a set of grouped line/box plots, especially when multiple interacting features are involved. A brief introduction to decision tree models is given below, and interested readers may refer to the book written by Breiman and colleagues for more information.^33^

By applying a set of splitting rules on the included features/predictors (e.g. deciding between sample size = 100 or 400), a decision tree recursively partitions the source data set (i.e. the tree's root) into non-overlapping subsets with supposedly homogenous cases inside each subset. The splitting rules are typically optimized by minimizing a heterogeneity/impurity statistic, such as the mean square error or Gini impurity associated with the model predictions. Once the optimal splitting rules are identified, final predictions are made by either averaging the values of a continuous outcome or selecting the majority class of a categorical outcome inside the terminal subsets (i.e. the leaf nodes that have no further split). The size of a tree is controlled by some predefined hyperparameters, such as the complexity parameter which sets the minimum improvement (e.g. reduction in mean square error) required to perform splits, the maximum number of splits performed by each tree, and/or the minimum number of cases required in each subset before/after performing each split. Thus, the final decision tree model will typically only retain the most influential features that result in the greatest improvement in prediction accuracy (e.g. the largest reduction in mean square error). When aiming for the best prediction accuracy, these hyperparameters can be optimally tuned through a full Cartesian grid search.

In the current study, instead of aiming for the best accuracy, the decision tree model was mainly used as a tool for quickly identifying the most influential features and their interactions, and therefore the hyperparameters were manually tuned. Specifically, we restricted the maximum depth of a branch to four splits before reaching the terminal node/leaf, while also fixing the complexity parameter at zero which allowed a node to split no matter how small the reduction in mean square error was. These settings helped to achieve a balance between selecting only the most influential features and displaying the full trend/pattern (up to four splits) once a split occurred on an important feature. All other hyperparameters were kept at the default settings of the ‘rpart’ package in R.^34^ In addition, while plotting the decision trees, we included the calculated averages of the evaluated outcomes (Type I error, power, or MAE for effect size estimation) at each split, which could be helpful for comparing the differences at varying depths of the tree when a specific set of conditions were fulfilled/unfulfilled.

## Results

A preliminary decision tree based on the full Type I error summary table showed a clear separation in splitting patterns between conditions with and without DIF, which indicated a strong impact of DIF. This finding was not surprising given past findings on DIF's significant biasing effects on sum scores and latent trait scores.^19^ Thus, for clarity, we will split each section of the evaluated outcomes into two subsections based on the presence or absence of DIF.

### Type I error

#### DIF conditions

**Figure 1.**
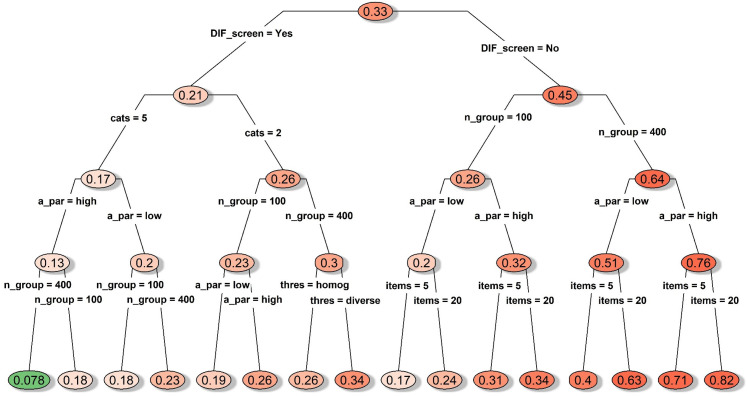
Decision tree for the average type I errors under conditions with simulated DIF item. *Notes*. A_par: discrimination parameter strength; cats: number of categories; DIF_screen: DIF prescreening; items: number of items; Method: type of mean comparison approach; missing: whether missing data were simulated; n_group: sample size per group; thres: whether the items had homogenous or diverse thresholds; DIF: differential item functioning.

When DIF items were present, all group mean comparison approaches had inflated Type I error, with a study-wise average Type I error of 0.33 (see the node on the top of Figure 1). Nonetheless, the average Type I error could still be reasonably controlled at 0.078 with values ranged between 0.05 and 0.12, when we performed DIF prescreening and had 400 subjects per group responding to highly discriminating polytomous items (see the cascading path leading to the leftmost node in Figure 1). If we further restrict the cases to conditions with homogenous item thresholds (not shown in Figure 1), Type I error could be reduced to an average of 0.069 with values ranged between 0.05 and 0.09. Differences across the three types of mean comparison approaches (sum-score, IRT-model, and IRT-EAP) were mostly negligible as long as the above favorable conditions were met.

#### DIF-free conditions

**Figure 2.**
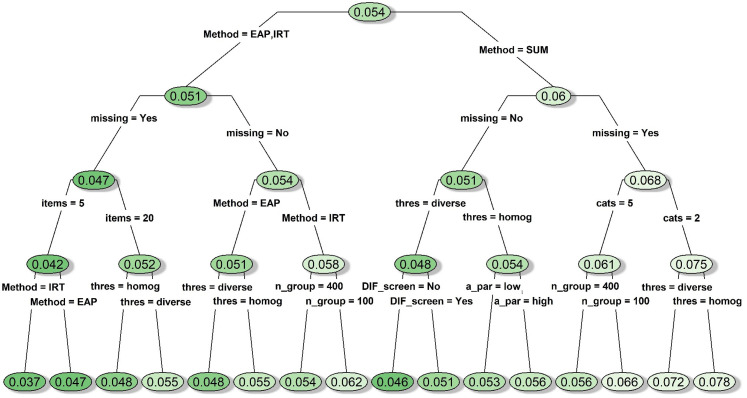
Decision tree for the average type I errors under conditions with no simulated DIF item. *Notes*. A_par: discrimination parameter strength; cats: number of categories; DIF_screen: DIF prescreening; items: number of items; Method: type of mean comparison approach; missing: whether missing data were simulated; n_group: sample size per group; thres: whether the items had homogenous or diverse thresholds; DIF: differential item functioning.

When DIF was not present, there were distinctive patterns in the average Type I errors across different types of mean comparison approaches. The average Type I errors associated with the sum-score approach were noticeably inflated when missing data were involved, reaching as high as 0.078 in some cases (see the rightmost node on Figure 2). This was likely due to bias caused by listwise deletion, and past simulation research has suggested that the results would improve if the sum-score approach was coupled with a better missing data handling technique such as multiple imputations.^11^ In contrast, most of the conditions associated with the IRT-model and IRT-EAP approaches (i.e. the left side of Figure 2) had Type I errors fluctuating around 0.05, with IRT-EAP yielding more consistent results. Type I error associated with the IRT-model approach had a slightly wider variation depending on the presence of missing data, number of items, and sample size.

### Statistical power

#### DIF conditions

**Figure 3.**
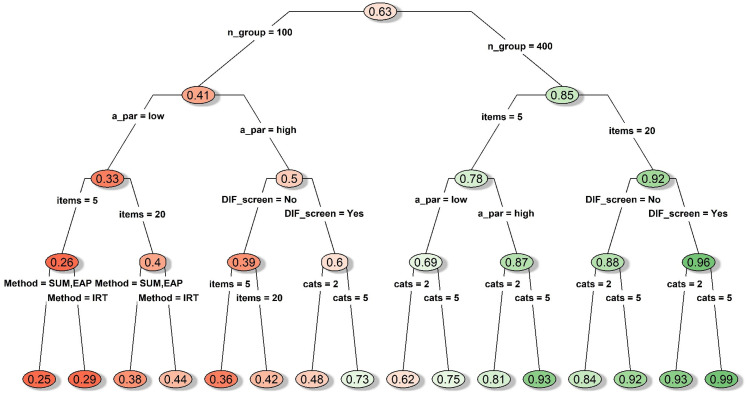
Decision tree for average statistical power under conditions with simulated DIF items. *Notes*. A_par: discrimination parameter strength; cats: number of categories; DIF_screen: DIF prescreening; items: number of items; Method: type of mean comparison approach; missing: whether missing data were simulated; n_group: sample size per group; thres: whether the items had homogenous or diverse thresholds; DIF: differential item functioning.

As shown in Figure 3, statistical power was not a concern when the sample size is large, except when all other features were in unfavorable conditions (five dichotomous items with low discriminations). Nonetheless, many of the conditions achieved high statistical power at the expense of highly inflated Type I error as discussed above. Thus, we will focus on statistical power for the specific set of conditions that yielded reasonably controlled Type I error. Unsurprisingly, statistical power across all three group comparison approaches was maintained at 100% when the favorable conditions were met (400 subjects per group responding to highly discriminating polytomous items with homogenous thresholds, and DIF prescreening was performed).

#### DIF-free conditions

**Figure 4.**
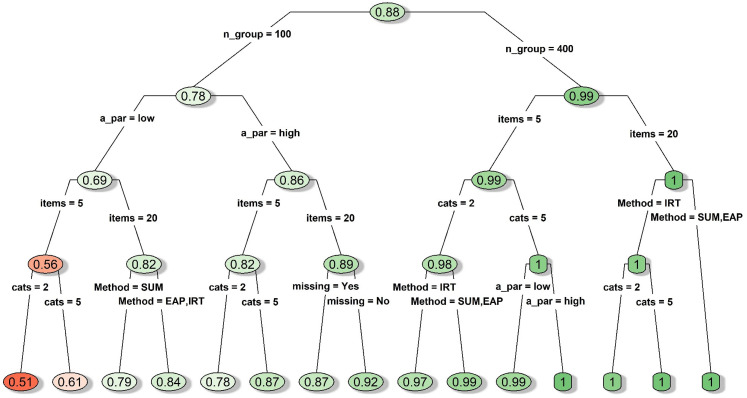
Decision tree for average statistical power under conditions with no simulated DIF items. *Notes*. A_par: discrimination parameter strength; cats: number of categories; DIF_screen: DIF prescreening; items: number of items; Method: type of mean comparison approach; missing: whether missing data were simulated; n_group: sample size per group; thres: whether the items had homogenous or diverse thresholds; DIF: differential item functioning.

As shown in Figure 4, in general, having a larger sample size, a longer measure, more response categories, items with high discrimination, or no missing data seemed to improve power under the DIF-free conditions.

When the sample size was 400 per group, power remained very high (average power = 0.99; see the right node after the first split in Figure 4), although the IRT-model approach showed relatively lower power when the measure contained only five dichotomous items (average power = 0.97; as shown on Figure 4).

When sample size was 100 per group, the average power dropped to as low as 0.51 when we only had five dichotomous items with low discriminations (see the leftmost node on Figure 4). However, by examining the full summary table more closely under the guidance of the decision tree model, we found that the lower bounds of power could be elevated to a satisfactory level (0.74 for sum-score, 0.84 for IRT-EAP, 0.82 for IRT-model) if any two of the three favorable conditions were met: i) having 20 items; ii) having highly discriminating items; iii) having polytomous items. Meeting all three conditions could bring the lower bound of power to 0.83 for the sum-score approach and 0.92 for the IRT-model and IRT-EAP approaches.

### Bias in effect size estimation

#### DIF conditions

**Figure 5.**
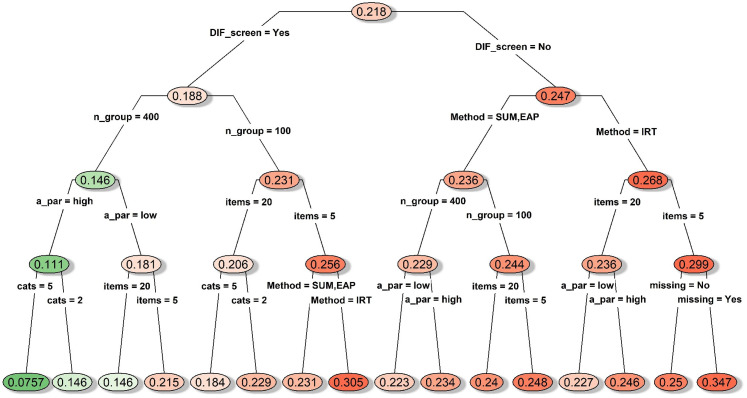
Decision tree for mean absolute error of the effect size estimates under conditions with simulated DIF item. *Notes*. A_par: discrimination parameter strength; cats: number of categories; DIF_screen: DIF prescreening; items: number of items; Method: type of mean comparison approach; missing: whether missing data were simulated; n_group: sample size per group; thres: whether the items had homogenous or diverse thresholds; DIF: differential item functioning.

A decision tree is displayed in Figure 5 for the MAE statistic under conditions with simulated DIF items. As expected, having features with favorable conditions (the implementation of DIF prescreening, a larger sample size, higher item discriminations, more response categories, and/or no missing data) generally led to a lower MAE, and the most accurate effect size estimates were obtained using 400 subjects per group taking highly discriminating polytomous items that were also prescreened for DIF (average MAE = 0.076 with values ranged between 0.062 and 0.130; see the leftmost node on Figure 5). However, it was quite interesting that having higher item discriminations was somewhat detrimental if DIF prescreening was skipped while having either a larger sample or a longer measure (see the right side of Figure 5), which implied interactions between these features. Although the three group mean comparison approaches did not differ much in terms of MAE under most DIF conditions, the worst MAE values were associated with the IRT-model approach, especially if DIF prescreening was skipped while having only five items with missing data (average MAE was 0.347 with values ranged between 0.302 and 0.431; see the rightmost node on Figure 5). Even if items have been prescreened for DIF, the IRT-model approach still performed worse than the other two approaches when a small sample size was combined with a short measure (average MAE was 0.305 with values ranged between 0.302 and 0.431; see the terminal node located in the middle of Figure 5).

Furthermore, when focusing on the conditions that yielded the overall best power and Type I error combination (400 subjects per group responding to highly discriminating polytomous items with homogenous thresholds, and DIF prescreening was performed), the average MAE was 0.074 with values ranged between 0.062 and 0.112. The only time that MAE inflated above 0.08 was when we carried out the IRT-model approach using five items with missing data.

#### DIF-free conditions

**Figure 6.**
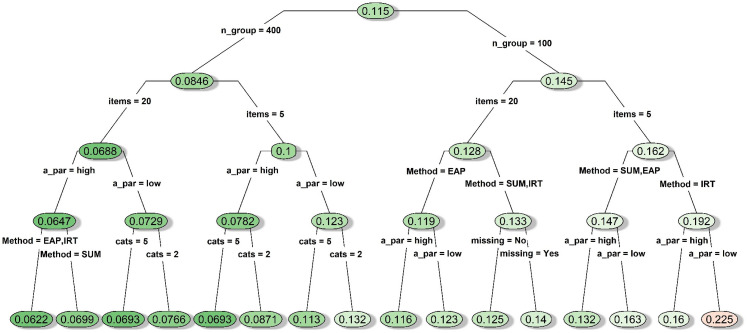
Decision tree for mean absolute error of the effect size estimates under conditions with no simulated DIF item. *Notes*. A_par: discrimination parameter strength; cats: number of categories; DIF_screen: DIF prescreening; items: number of items; Method: type of mean comparison approach; missing: whether missing data were simulated; n_group: sample size per group; thres: whether the items had homogenous or diverse thresholds; DIF: differential item functioning.

A decision tree is displayed in Figure 6 for the MAE statistic under DIF-free conditions. Not surprisingly, the MAE values under the DIF-free conditions were generally lower than those observed under the DIF conditions, and whether we had prescreened the items for DIF was no longer an influential feature. As expected, the bias in effect size estimation was relatively lower when some favorable conditions were met (e.g. a larger sample, a longer measure, more response categories, high-discrimination parameters, and/or no missing data). MAE was overall much lower when the sample size was 400 per group with an average MAE at 0.0846. When the sample size was 100 per group, the average MAE was 0.145, which never dropped below 0.112 and could be inflated to as high as 0.293. The IRT-EAP approach had the best performance when the sample size was small, especially when we had 20 items. The worst MAE under the DIF-free conditions was associated with the IRT-model approach when we had a small sample, a short measure, and items with low discriminations (see the rightmost node in Figure 6).

## Discussion

The current study extended the work of previous simulations on the performance of different group mean comparison methods (sum-score, IRT-model, and IRT-EAP), by incorporating four new manipulated features (presence/absence of DIF items, two/five-item categories, high/low item discriminations, homogenous/diverse item thresholds). The results overall supported our hypotheses that statistical power, Type I error, and effect size estimation associated with group mean comparison are impacted by the newly investigated features and their interactions with other manipulated features including the type of mean comparison approach being implemented. It is worth noting that, among the four newly investigated features, item thresholds had the least impact on group mean comparison results, given that it rarely showed up in the decision tree models and the three outcome criteria did not change much depending on properties of the thresholds.

### Findings

In the ideal scenario where no DIF is involved, consistent with past research findings, group mean comparisons based on all three approaches showed similar levels of Type I error, power, and effect size estimation accuracy in most conditions (see the Results section, and Figures 2, 4 and 6). Furthermore, having a smaller sample size and/or a shorter measure had a stronger negative impact on the performance of the IRT-model approach, while missing data (with listwise deletion) had a stronger negative impact on the sum-score approach. These findings were also consistent with extant literature.^10–13^ Across all three mean comparison approaches, having a larger sample size, a longer measure, more response categories, higher discrimination, and no missing data generally resulted in higher power and more accurate effect size estimation. Type I errors associated with the IRT-model and IRT-EAP approaches were quite consistent regardless of simulation conditions, whereas the sum-score approach's Type I error could be noticeably inflated when missing data were present (see Figure 2).

In the more realistic scenario where some DIF items existed,^15,16^ group mean comparisons based on all three approaches showed highly inflated Type I errors, even though having some features with favorable conditions (i.e. 400 subjects per group responding to highly discriminating polytomous items with homogenous thresholds, and DIF prescreening was performed) could help control Type I error below 0.09 across the board. When these favorable conditions were met, power was also very high and effect size estimation was quite accurate across all three approaches. Nevertheless, comparing to the other two approaches, the IRT-model approach was somewhat inconsistent in estimating effect sizes in some cases, with the average MAEs reaching above 0.25 (which was equivalent to a 50% deviation from the true value of 0.50; see Figure 5).

### Limitations

Although the current study incorporated features that have not been investigated in previous simulation studies, only a limited number of conditions within each feature were examined (e.g. high vs. low item discrimination). Also, to maintain a manageable number of simulation conditions, several potentially influential features had fixed values (e.g. having 40% directional DIF items). In addition, means were compared for only two groups. Due to these limitations, our results cannot be generalized to all applied settings, and therefore additional investigations are needed in the future.

Also, our results could possibly be influenced in part by the DIF prescreening procedure, since only the IRT likelihood ratio test was performed during anchor selection and DIF testing. Even though the IRT likelihood ratio test is an excellent tool for anchor selection and DIF detection purposes, it might not be the ideal method under conditions with small sample sizes and/or shorter measures.^24^ A discussion on the most appropriate DIF detection method under different scenarios is beyond the scope of the current study. Interested readers may refer to external resources for comparisons of different DIF detection methods.^35^

In addition, the IRT-EAP approach being applied in the current study assumed that item parameters were pre-calibrated with small errors (±0.2 around the true values). Such a specification might have given the IRT-EAP approach some advantages over the other two approaches. It is also worth pointing out that the accuracy of such a model calibration also depends on how closely the prior distribution (see Equation 6 in the Method section) matches the true person parameter distribution. When the underlying distribution is suspected to be nonnormal, alternative methods (e.g. the Ramsay or Davidian curve approach) shall be considered for better item parameter and score estimation accuracy.^36,37^ Nonetheless, previous findings suggested that statistical power associated with group mean comparisons is unaffected regardless of whether item parameters are perfectly calibrated.^10^

### Recommendations for applied researchers

In situations where a well-established PROs measure has been thoroughly evaluated in large representative samples and DIF has not been found (or mostly eliminated) between the groups being compared, researchers’ best choice would be adopting the IRT-EAP approach which shows more consistent performance across various conditions. On one hand, unlike the sum-score approach, the IRT-EAP approach is less affected by missing data. On the other hand, the IRT-EAP approach is more robust than the IRT-model approach when the sample size is small and/or the measure is short.

For a measure still at its development stage with suspected DIF items, the best strategy is to run a DIF prescreening procedure before group means are compared. Even though the anchor selection and DIF detection strategies based on the IRT likelihood ratio test adopted for the current study are recommended by many researchers for its overall good performance,^24,32,38^ one may also consider alternative DIF prescreening methods for cross-validation purposes.^26,39,40^ Moreover, to achieve better Type I error control and more accurate effect size estimation, it would be quite helpful to recruit a larger sample (e.g. 400 per group) and to implement high-quality polytomous items especially those with high discriminations. The IRT-EAP approach remains as the best choice when DIF items have been detected and controlled, followed by the sum-score approach.

Researchers working on power or sample size calculations for study designs that are similar to the simulated conditions in the current study may refer to the full summary tables in the online supplemental materials section for the expected statistical power and Type I error.

### Conclusions

Group mean comparisons based on all three approaches (sum-score, IRT-model, and IRT-EAP) will work similarly well when certain favorable conditions (e.g. a large sample size, a longer measure, more response categories, high item discriminations) are met and all items are prescreened for suspected DIF. The IRT-EAP approach yields the most consistent results and is less affected by small sample sizes, shorter measures, or missing data. In many cases, the simple sum-score approach should also yield results comparable to those obtained using IRT-EAP. Given that the IRT-model approach requires additional modeling fitting and does not show a clear advantage over the other two approaches, we do not recommend the use of the IRT-model approach for group mean comparisons in most circumstances.

## Supplemental Material

sj-xlsx-1-smm-10.1177_09622802211043263 - Supplemental material for Evaluations of the sum-score-based and item response theory-based tests of group mean differences under various simulation conditions Click here for additional data file. Supplemental material, sj-xlsx-1-smm-10.1177_09622802211043263 for Evaluations of the sum-score-based and item response theory-based tests of group mean differences under various simulation conditions by Mian Wang and Bryce B. Reeve in Statistical Methods in Medical Research

sj-xlsx-2-smm-10.1177_09622802211043263 - Supplemental material for Evaluations of the sum-score-based and item response theory-based tests of group mean differences under various simulation conditions Click here for additional data file. Supplemental material, sj-xlsx-2-smm-10.1177_09622802211043263 for Evaluations of the sum-score-based and item response theory-based tests of group mean differences under various simulation conditions by Mian Wang and Bryce B. Reeve in Statistical Methods in Medical Research

sj-xlsx-3-smm-10.1177_09622802211043263 - Supplemental material for Evaluations of the sum-score-based and item response theory-based tests of group mean differences under various simulation conditions Click here for additional data file. Supplemental material, sj-xlsx-3-smm-10.1177_09622802211043263 for Evaluations of the sum-score-based and item response theory-based tests of group mean differences under various simulation conditions by Mian Wang and Bryce B. Reeve in Statistical Methods in Medical Research

## Appendix

## R code for generating the item pool of 10,000 five- and two-category graded response items ##

########################

# Preparation

########################

library(truncnorm) #Load necessary packages

library(plyr) #Load necessary packages

nitems <−10000 #Set the number of items to be generated

set.seed(20170616) #Set a seed for the random number generator

#######################

# Item with five categories

#######################

#Discrimination parameter

a1.high <−runif(n = nitems/2, min = 1.7, max = 3.5)

a1.low <−runif(n = nitems/2, min = 0.5, max = 1.0)

#Location parameter

b1 <−rtruncnorm(n = nitems, a = −3.5, b = 0.5, mean = −1, sd = 1)

b2 <−b1 + rtruncnorm(n = nitems, a = 0.4, b = 1.2, mean = 0.8, sd = 0.2)

b3 <−b2 + rtruncnorm(n = nitems, a = 0.4, b = 1.2, mean = 0.8, sd = 0.2)

b4 <−b3 + rtruncnorm(n = nitems, a = 0.4, b = 1.2, mean = 0.8, sd = 0.2)

#Construct a dataframe

sim.pars.cat5 <− data.frame(c(a1.high, a1.low), cbind(b1, b2, b3, b4))

colnames(sim.pars.cat5) <− c(“a1”, paste0(“b”, 1:4))

#Label for discrimination magnitude

sim.pars.cat5$discmag <− c(rep(“high”, nitems/2), rep(“low”, nitems/2))

#######################

#Item with two categories

#######################

#Discrimination parameter

a_dich.high <− runif(n = nitems/2, min = 1.7, max = 3.5)

a_dich.low <− runif(n = nitems/2, min = 0.5, max = 1.0)

#Location parameter

b_dich <− rtruncnorm(n = nitems, a = −2.5, b = 2.4, mean = 0.1, sd = 1.3)

#Construct a dataframe

sim.pars.cat2 <− data.frame(c(a_dich.high, a_dich.low), b_dich)

colnames(sim.pars.cat2) <− c(“a1”, “b1”)

#Label for discrimination magnitude

sim.pars.cat2$discmag <− c(rep(“high”, nitems/2), rep(“low”, nitems/2))
